# No impact of COVID-19 at delivery on maternal mortality or infant adverse birth outcomes in Botswana during the Omicron era

**DOI:** 10.1371/journal.pone.0310980

**Published:** 2024-09-25

**Authors:** Jaspreet Banga, Maya Jackson-Gibson, Modiegi Diseko, Ellen C. Caniglia, Gloria Mayondi, Judith Mabuta, Rebecca Luckett, Sikhulile Moyo, Pamela Smith-Lawrence, Mosepele Mosepele, Shahin Lockman, Joseph Makhema, Rebecca Zash, Roger Shapiro

**Affiliations:** 1 Division of Infectious Diseases, Beth Israel Deaconess Medical Center, Boston, Massachusetts, United States of America; 2 Department of Pediatrics, Children’s Hospital of Philadelphia, Philadelphia, Pennsylvania, United States of America; 3 Botswana Harvard Health Partnership, Gaborone, Botswana; 4 Perelman School of Medicine, University of Pennsylvania, Philadelphia, Pennsylvania, United States of America; 5 Department of Obstetrics and Gynecology, Beth Israel Deaconess Medical Center, Boston, Massachusetts, United States of America; 6 Department of Immunology and Infectious Diseases, Harvard T.H. Chan School of Public Health, Boston, Massachusetts, United States of America; 7 Ministry of Health and Wellness, Gaborone, Botswana; 8 Division of Infectious Diseases, Brigham and Women’s Hospital, Boston, Massachusetts, United States of America; CEA, FRANCE

## Abstract

SARS-CoV-2 infection during pregnancy was associated with maternal mortality and adverse birth outcomes in the pre-Omicron era, including a stillbirth rate of 5.6% in Botswana. We re-evaluated these outcomes in the Tsepamo Study during the Omicron era. We assessed maternal mortality and adverse birth outcomes for all singleton pregnancies from mid-November 2021 (the start of the Omicron era) to mid-August 2022 at nine Tsepamo sites, among individuals with documented SARS-CoV-2 screening PCR or antigen tests and known HIV status. Of 9,705 women routinely screened for SARS-CoV-2 infection at delivery (64% of deliveries at these sites), 373 (3.8%) tested positive. Women with HIV were as likely to test positive for SARS-CoV-2 (77/1833, 4.2%) as women without HIV (293/6981, 4.2%) (p = 1.0). There were 5 recorded maternal deaths (0.03%), one occurring in a woman with a positive SARS-CoV-2 test result. In contrast, maternal mortality was 3.7% and 0.1% in those with and without SARS-CoV-2, respectively, during the pre-Omicron era. In the Omicron era, there were no differences among infants exposed or unexposed to SARS-CoV-2 in overall adverse birth outcomes (28.1% vs 29.6%; aRR 1.0, 95%CI 0.8–1.1), severe adverse birth outcomes (11.9 vs 10.6%; aRR 1.1, 95%CI 0.8–1.5), preterm delivery (15.1% vs 14.9%; aRR 1.0, 95%CI 0.8–1.3), or stillbirth (1.9% vs 2.3%; aRR 0.8, 95%CI 0.4–1.7). Adverse outcomes among those exposed to both HIV and SARS-CoV-2 were similar to those exposed to HIV alone (31.2% vs. 33.1%; aRR 0.9, 95%CI 0.6–1.3; p = 0.5). Maternal mortality was far lower in Botswana during the Omicron era than in the pre-Omicron era, and adverse birth outcomes were no longer significantly impacted by exposure to SARS-CoV-2 either overall or with HIV co-exposure. Increased population immunity to SARS-CoV-2, less stress on the hospital systems in the Omicron era, and possible differences in viral pathogenicity may combine to explain these findings.

## Introduction

COVID-19 may impact both maternal mortality and adverse birth outcomes globally, especially in less resourced regions [1, 2]. Few studies have evaluated whether COVID-19 differentially impacts these outcomes for people living with HIV (PLHIV) and their children [3–7]. Prior data from Botswana, where approximately 21% of deliveries occur among PLHIV, showed that active COVID-19 near delivery was associated with high maternal mortality and a concerning overall stillbirth prevalence of 5.6%. While these outcomes did not differ by maternal HIV status, most other adverse birth outcomes were more severe among infants of PLHIV, consistent with prior data, though seemingly proportionally increased in neonates exposed to HIV and SARS-CoV-2. Additionally, pregnant PLHIV were more likely to test positive for COVID-19 at delivery [8]. However, this study was conducted during the initial COVID-19 waves in Botswana in 2020–2021, when Beta and then Delta variants of SARS-CoV-2 were circulating, healthcare resources were strained, and vaccine access and immune recognition of SARS-CoV-2 from prior infections were limited.

Between November 2021 and August 2022, Botswana was impacted by the Omicron variant of SARS-CoV-2, a variant that has been associated with increased transmissibility and decreased mortality. There are limited data to date on adverse birth outcomes in the Omicron era, especially by HIV status and in countries with high HIV prevalence [7, 9–11]. To better understand maternal and infant outcomes in the Omicron era, we performed an updated analysis at similar surveillance sites in Botswana as the initial 2020–2021 study, and stratified results by maternal HIV status.

## Methods

Methods for data collection and analysis have largely been previously described in the published pre-Omicron era analysis, and are summarized here along with any changes specific to this analysis [8]. Data for this retrospective analysis were collected from the ongoing Tsepamo Study, which has been performing birth outcomes surveillance in Botswana at up to 18 public hospitals, covering approximately 66% of all births in the country, since 2014 [12]. The Tsepamo Study was originally designed to evaluate the association between HIV, antiretroviral therapy (ART) and adverse birth outcomes, but also began capturing COVID-19 status at delivery in 2020 as routine SARS-CoV-2 testing of all admitted pregnant patients was rolled out in government maternity wards [13]. This screening was routinely performed in 2021–2022, but declined by late 2022 and the timing of decline varied by maternity site.

The current analysis includes nine Tsepamo study sites that were performing routine COVID-19 screening with >50% of delivering women screened for SARS-CoV-2 upon maternity ward admission from November 15, 2021 (the start of the Omicron era) through August 15, 2022. Although testing continued beyond this period, positivity was low, and routine testing dropped to <50% of all deliveries, making it less likely to be representative of all delivering women than during the study period chosen for analysis. Four of the 13 sites included in the prior Botswana analysis were excluded for low COVID-19 screening rates in the new study period [8]. COVID-19 screening was performed by nasal swabs as part of routine care by government medical teams, using either PCR testing (2019-nCoV RNA PCR Fluorescence Probing Assay, Sun Yat-sen University, Da An Gene Co.—sensitivity/specificity 68.4%/100%) or rapid antigen testing (Abbott PanBio–sensitivity/specificity 98.1%/99.8%—or the SD Biosensor Standard Q Covid Ag–sensitivity/specificity 94.9%/100%) [14–16]. During the study period, the majority of COVID-19 testing was performed by rapid antigen testing based on test availability, but the exact percentages were not documented. Data were deidentified and authors could not identify individual participants during or after data collection.

This analysis includes all in-hospital singleton deliveries with known maternal HIV status and a COVID-19 screening test performed no more than 14 days before and up to 3 days after delivery. We defined individuals as positive with SARS-CoV-2 if they had any positive test during this window period and individuals were considered negative for SARS-CoV-2 if they had a negative test within that same time period. All others (indeterminate, pending, unknown or no documented test results) were defined as COVID-19 status unknown and excluded from the analysis. Individuals who delivered twins or triplets were excluded because of the known increased risk of birth complications with multiple gestations [17]. Data were not collected for deliveries that occurred prior to arriving to the hospital, infants transferred from a different hospital after delivery, or deliveries before 24 weeks gestational age (classified as miscarriage in Botswana). Outcomes of interest were maternal mortality and adverse birth outcomes. We defined maternal mortality as all in-hospital deaths on the maternity ward after delivery. Any adverse birth outcome included preterm delivery, small for gestational age (SGA), stillbirth, or neonatal death. Any severe adverse birth outcome included very preterm delivery, very small for gestational age, stillbirth, or neonatal death. Definitions of preterm delivery, SGA, very small for gestational age, stillbirth, and neonatal death were as previously described [8].

We first evaluated associations between maternal COVID-19 status at delivery and maternal and birth outcomes, and then subsequently stratified our analyses by maternal HIV status. Log binomial regression was performed to estimate risk ratios for each outcome by COVID-19 status at delivery overall and stratified by HIV status. In multivariate models, we adjusted for age, employment, delivery at referral hospital and HIV status. These factors were chosen a priori based on factors associated with adverse birth outcomes in prior studies in Botswana, and factors associated with severe COVID-19 [12, 18]. A multivariate analysis was performed to evaluate for potential confounding in the study. We then conducted similar analyses evaluating associations between maternal HIV status at delivery and maternal and birth outcomes, stratifying by COVID-19 status. We have followed STROBE guidelines for the reporting of our analysis and statistical analyses were performed using SAS version 9.4.

Institutional approval and waiver of individual and minor consent for the Tsepamo Study surveillance were granted by the Health Research and Development Committee in Botswana and by the Office of Regulatory Affairs and Research Compliance at the Harvard T. H. Chan School of Public Health in Boston, Massachusetts. Additional information regarding the ethical, cultural, and scientific considerations specific to inclusivity in global research is included in the Supporting Information [S1 File].

## Results

We recorded a total of 15,101 individuals with a singleton delivery at included surveillance sites during the study period. We excluded 6,272 (42%) without documented COVID-19 screening and an additional 15 (0.2%) without documented HIV status. This analysis includes 8,814 (58%) who were routinely screened for COVID-19 around the time of delivery. Among the included population, 370 (4%) had a positive SARS-CoV-2 antigen or PCR test at delivery, of which 77 (21%) were also living with HIV. People living with HIV were equally likely to test positive for SARS-CoV-2 (4.20%, p = 1.0).

Baseline characteristics by COVID-19 status at time of delivery are shown in Table 1. Individuals who tested positive for COVID-19 had similar age, educational status, employment status, and primigravity compared with those who tested negative for COVID-19. Based on data abstracted from maternity cards, there was no difference in SARS-CoV-2 positivity at delivery by pre-existing diagnoses of cardiac disease, diabetes, chronic hypertension, or asthma. A larger portion of individuals who tested positive for SARS-CoV-2 delivered at a referral hospital, which are also urban sites. COVID-19 vaccination status was not known for individuals; vaccination with one of several vaccines became available in Botswana starting in March 2021 and recommended for pregnant women in December 2022. By the end of our study period, it is estimated that the majority of pregnant women were vaccinated against COVID-19.

**Table 1:** Baseline characteristics by COVID status.

	COVID-19 + (N = 370)	COVID-19 - (N = 8,444)
**Maternal Age, median [IQR]** ^**a**^	27 (IQR 22–33)	27 (IQR 21–33)
**No/primary education** ^**b**^	20 (5.5%)	577 (6.9%)
**Delivered at referral hospital**	241 (65.1%)	3889 (46.1%)
**Unemployed** ^**c**^	204 (57.6%)	4863 (59.6%)
**Primigravid** ^**d**^	122 (33.0%)	3029 (35.9%)
**Past medical history of**:		
** Cardiac disease**	2 (0.5%)	24 (0.3%)
** Pre-gestational diabetes**	3 (0.8%)	40 (0.5%)
** Chronic hypertension**	18 (4.9%)	239 (2.8%)
** Asthma**	7 (1.9%)	223 (2.6%)

^a^ missing 1 COVID+ value and 8 COVID- values

^b^ missing 9 COVID+ values and 135 COVID- values

^c^ missing 16 COVID+ values and 280 COVID- values

^d^ missing 15 COVID- values

Infants born to individuals with COVID-19 at time of delivery had similar risk of adverse birth outcomes (RR 0.9, 95% CI 0.8–1.1), any severe adverse birth outcomes (RR 1.1, 95% CI 0.8–1.5), preterm delivery (RR 1.0, 95% CI 0.8–1.3), very preterm delivery (RR 0.9, 95% CI 0.5–1.6), small for gestational age (RR 0.9, 95% CI 0.7–1.1), very small for gestational age (RR 1.1, 95% CI 0.8–1.7), stillbirth (RR 0.8, 95% CI 0.4–1.7), and neonatal death (RR 1.3, 95% CI 0.6–2.7) as those born to individuals without COVID-19 at delivery. There were a total of 35 (0.4%) maternal intensive care unit admissions and 5 (0.06%) maternal deaths recorded among those included in the analysis during the study period, one occurring in a patient with COVID-19. A comparison of adverse birth outcomes in the pre-Omicron and Omicron eras from this population in Botswana is shown in Table 2.

**Table 2:** Adverse birth outcomes in pre-Omicron era vs Omicron era.

Adverse birth outcome	Pre-Omicron era N = 11,483				Omicron era N = 8,814			
	HIV+ COVID- (N = 2,277)	HIV- COVID+ (N = 395)	HIV+ COVID+ (N = 144)	HIV- COVID- (N = 8,667)	HIV+ COVID- (N = 1,756)	HIV-COVID+ (N = 293)	HIV+ COVID+ (N = 77)	HIV-COVID- (N = 6,688)
**Stillbirth**	69 (3.0%)	24 (6.1%)	6 (4.2%)	228 (2.6%)	52 (3%)	7 (2.4%)	0	146 (1.7%)
**Preterm delivery**	326 (14.7%)	76 (19.8%)	36 (25.5%)	1105 (13.1%)	282 (16.5%)	42 (14.6%)	13 (17.1%)	950 (14.5%)
**Very preterm delivery**	65 (2.9%)	14 (3.7%)	9 (6.4%)	256 (3.0%)	68 (4%)	8 (2.8%)	4 (5.3%)	224 (3.4%)
**Small for gestational age (SGA)**	405 (18.3%)	51 (13.5%)	31 (22.0%)	1211 (14.4%)	319 (18.8%)	40 (14%)	11 (14.7%)	1036 (15.9%)
**Very SGA**	160 (7.2%)	17 (4.5%)	12 (8.5%)	449 (5.3%)	130 (7.7%)	19 (6.6%)	6 (8%)	366 (5.6%)
**Neonatal death**	33 (1.5%)	6 (1.6%)	4 (2.9%)	105 (1.3%)	34 (2%)	6 (2.1%)	1 (1.3%)	90 (1.4%)

**Fig 1:**
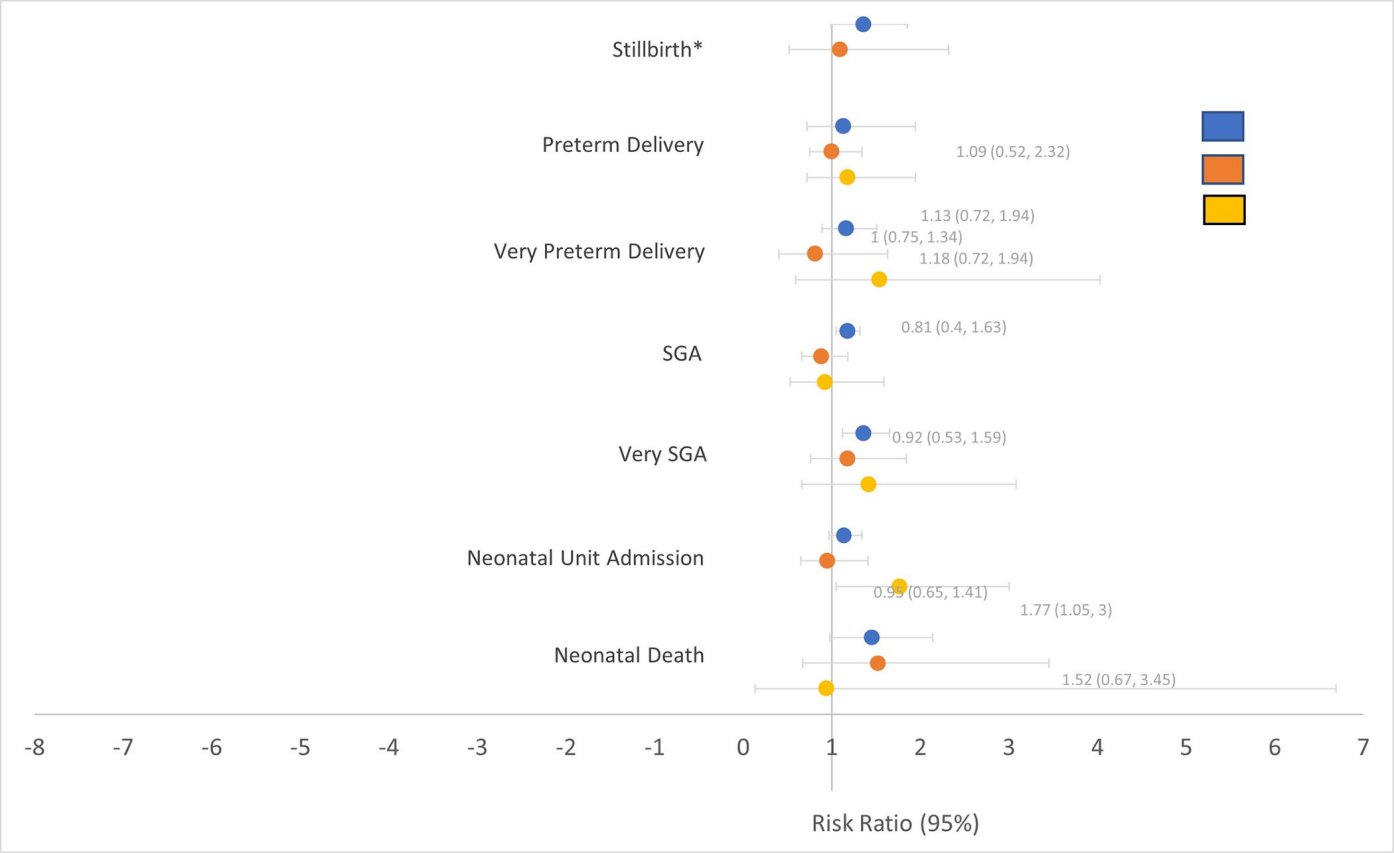
Risk ratios of adverse birth outcomes by COVID-19 and HIV status, compared with infants unexposed to either COVID-19 or HIV.

Infants born to PLHIV had significantly more adverse birth outcomes overall compared with infants born to HIV-negative mothers (32.5% vs 27.7%; RR 1.17, 95% CI 1.12–1.23, p<0.0001), as expected from prior Tsepamo analyses; this difference remained after adjusting for COVID-19 at time of delivery (RR 1.15, 95% CI 1.07–1.24, p = 0.0002). Similarly, adverse birth outcomes among those exposed to both HIV and SARS-CoV-2 were similar to those exposed to HIV alone (31.2% vs 33.1%; RR 0.9, 95% CI 0.7–1.3). There were no significant differences in stillbirth, preterm delivery, very preterm delivery, SGA, very SGA, neonatal unit admission, and neonatal death when stratified by HIV and COVID status at time of delivery (Fig 1).

## Discussion

A COVID-19 diagnosis near delivery has been a major health concern since the beginning of the pandemic, with several studies–including surveillance from the Tsepamo dataset in Botswana—reporting increased risk of severe maternal morbidity and adverse birth outcomes [4, 8, 19–21]. In contrast to these early studies, we now report a decreased maternal mortality and no detectable increase in infant adverse birth outcomes in Botswana during the Omicron era, both overall and for PLHIV.

Our results are consistent with prior studies describing increased risk of adverse maternal and birth outcomes with COVID-19, though with decreased severity in the Omicron era [7, 22]. A population-based cohort study in Scotland assessed maternal and infant outcomes in women with COVID-19 during the Omicron and Delta waves and, similarly to our findings, found that outcomes had worsened during the Delta era, but most improved during the Omicron era [7]. A prospective study in an urban setting also found increased illness severity with the Delta variant as compared with Omicron after adjusting for prior vaccination [23]. In contrast, a recent study conducted by the WHO Global Clinical Platform for COVID-19 found only modest reduction in mortality risk with Omicron compared to Delta infection among PLHIV, though this did not specifically comment on pregnant populations and cited risk factors of low CD4 and older age [24]. Additionally, a multinational, observational study reported that COVID-19 in pregnancy was still associated with an increased risk of maternal morbidity and severe complications—particularly among symptomatic and unvaccinated women—during the first 6 months after Omicron was declared a variant of concern [25]. This latter study suggests that disease severity overall, perhaps mediated by immune exposure from prior infection or vaccination, may be an important driver of outcomes.

Our maternal and birth outcomes findings are consistent with more favorable outcomes in general during the Omicron wave of the epidemic, and we believe the reasons may be multifactorial [7, 22, 23]. Possible explanations include increased population immunity to SARS-CoV-2 through natural infection and vaccination; less stress on the hospital system in Botswana during the Omicron era, in contrast to the Delta wave in 2021 when hospitals were overburdened; and potential differences in viral pathogenicity that may be independent of these other factors [26–29]. To our knowledge, our study reports outcomes on the largest number of PLHIV and their infants at the time of a COVID-19 diagnosis at delivery, and it is reassuring that prior disparities by HIV status are no longer apparent.

We found that PLHIV had similar risk for adverse birth outcomes and maternal complications regardless of their COVID-19 status during the Omicron era, which differed from findings from Tsepamo data in prior eras. Prior Tsepamo data found that infants exposed to both COVID-19 and HIV had the highest prevalence of most adverse birth outcomes, and also demonstrated that PLHIV were more likely to have COVID-19 at delivery than those without HIV. Neither of these findings persisted in the Omicron era. Although infants exposed to HIV overall had a modest increase in adverse birth outcomes, these findings were expected and did not differ from prior Tsepamo analyses [8].

The strengths of this study include the large sample size and use of a representative population with known HIV status, delivering in government facilities that offered routine COVID-19 screening. Our study also had limitations. Data were only collected at time of delivery and therefore COVID-19 at any other time in pregnancy was not considered. We also did not have data on early pregnancy loss (before 24 weeks gestation) and therefore are unable to comment on any association between SARS-CoV-2 infection and miscarriages as these pregnancies ended before they could be included in our surveillance. Symptoms of COVID-19 were not included in the obstetric record and therefore we were unable to assess differences in outcome based on COVID-19 symptom severity, and do not know if symptomatic patients were preferentially screened at delivery. However, the proportion of those screened in this analysis (58%) is similar to the proportion screened in the previous analysis (56%). HIV viral load and CD4 count were not collected at the time of COVID-19 diagnosis. We also did not have viral sequencing and therefore the dominant variant in the wider population was used as a proxy for Omicron variant identification. Vaccination status was not available at the individual level, but could be inferred from population-level data. Because this study was observational, other residual confounding may also be present.

In conclusion, we demonstrated markedly decreased maternal mortality and stillbirths in Botswana during the Omicron era compared with the pre-Omicron era, and no association between COVID-19 and adverse birth outcomes, either overall or with HIV co-exposure. These findings are reassuring, and allow us to anticipate a low overall public health impact on maternal and infant outcomes from Omicron variants of SARS-CoV-2 as we enter the endemic phase of COVID-19 response.

## Supporting information

S1 File Inclusivity in global research. (DOCX)
